# Extracellular Biomarkers of Inner Ear Disease and Their Potential for Point‐of‐Care Diagnostics

**DOI:** 10.1002/advs.202104033

**Published:** 2021-12-26

**Authors:** Sahar Sadat Mahshid, Aliaa Monir Higazi, Jacqueline Michelle Ogier, Alain Dabdoub

**Affiliations:** ^1^ Biological Sciences Sunnybrook Research Institute Sunnybrook Health Sciences Centre Toronto ON M4N 3M5 Canada; ^2^ Department of Clinical and Chemical Pathology Minia University Minia 61519 Egypt; ^3^ Department of Otolaryngology–Head & Neck Surgery University of Toronto Toronto ON M5G 2C4 Canada; ^4^ Department of Laboratory Medicine and Pathobiology University of Toronto Toronto ON M5S 1A8 Canada

**Keywords:** biomarkers, cochlea, diagnosis, hair cells, hearing, inner ear, point‐of‐care

## Abstract

Rapid diagnostic testing has become a mainstay of patient care, using easily obtained samples such as blood or urine to facilitate sample analysis at the point‐of‐care. These tests rely on the detection of disease or organ‐specific biomarkers that have been well characterized for a particular disorder. Currently, there is no rapid diagnostic test for hearing loss, which is one of the most prevalent sensory disorders in the world. In this review, potential biomarkers for inner ear‐related disorders, their detection, and quantification in bodily fluids are described. The authors discuss lesion‐specific changes in cell‐free deoxyribonucleic acids (DNAs), micro‐ribonucleic acids (microRNAs), proteins, and metabolites, in addition to recent biosensor advances that may facilitate rapid and precise detection of these molecules. Ultimately, these biomarkers may be used to provide accurate diagnostics regarding the site of damage in the inner ear, providing practical information for individualized therapy and assessment of treatment efficacy in the future.

## Graphical abstract

Blood‐based biomarkers could precisely diagnose inner ear disorders in the future. The current lack of knowledge about specific biomarkers has been the main challenge for the development of blood‐based diagnostics. This review summerizes the current knowledge of inner ear biomarkers and how to effectively detect them, to facilitate the design of novel diagnostic strategies.

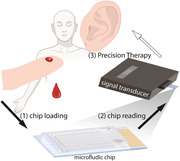

## 1 Introduction

Our ability to hear is one of the five fundamental human senses connecting us with our environment. Dysfunction within the auditory system causes hearing loss, communicative isolation, speech complications, poor social development, cognitive decline, and depression.^[^ ^1^ ^]^ The World Health Organization estimates that over 5% of the world's population has disabling hearing loss or deafness (https://www.who.int/news‐room/fact‐sheets/detail/deafness‐and‐hearing‐loss). As result, significant research efforts have focused on hearing preservation or regeneration. Yet, the current diagnostic pipeline for individuals with hearing loss is based on physical and neurological examinations performed by highly trained personnel using expensive, facility‐specific equipment, which rarely identifies site‐specific lesions (reviewed in ref. [2]). For example, the most utilized hearing test is the Auditory Brainstem Response (ABR), which is performed by a trained audiologist, with tailor‐made equipment that must be isolated from sound and electrical interference (reviewed in ref. [3]). Notably, ABR testing provides a quantitative measure of neuronal firing in the auditory pathway in response to short auditory stimuli, indicating that sound is being detected at a certain volume or frequency (known as a hearing threshold). However, the interpretation of ABR results is subjective and can only provide clues as to which aspect of the auditory pathway is dysfunctional in hearing‐impaired individuals. Therefore, in order to improve the outcome of diagnostics, effective biomarkers that report on the health or disease status of specific regions in the inner ear must be elucidated.^[^ ^4^ ^]^ Extracellular biomarkers are quantifiable indicators such as specific proteins, deoxyribonucleic and ribonucleic acids (DNAs and RNAs), or small molecules, which can be detected in easily accessible body fluids, like whole blood.^[^ ^5^ ^]^

**Figure 1:**
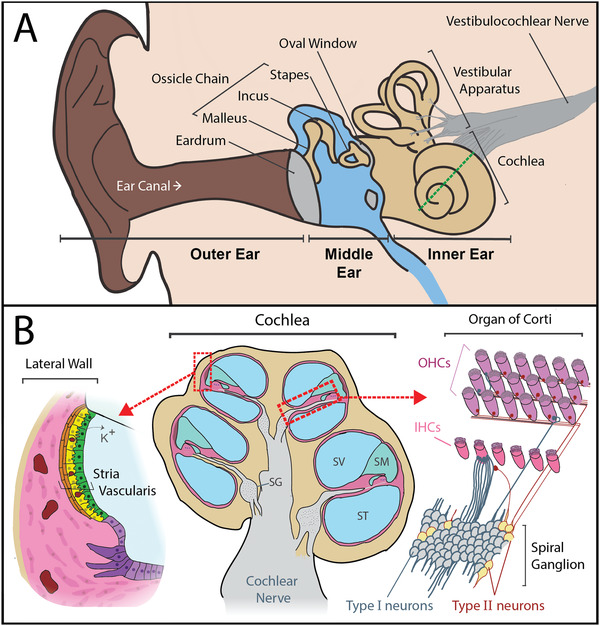
Anatomy of the ear and notable sites of degeneration that cause hearing loss. A) The outer ear collects and directs sound toward the tympanic membrane (eardrum). Soundwaves cause the eardrum to vibrate, and these vibrations are transferred to the bones of the ossicular chain (malleus, incus, and stapes) in the middle ear. This process amplifies the soundwaves. Amplified vibrations are then transferred to the inner ear, when the stapes footplate vibrates against the oval window of the cochlea. The inner ear consists of the cochlea and vestibular apparatus, where sound and movement are sensed respectively. The dotted green line represents the cochlear cross‐section displayed in (B). B) A cross‐section of the cochlea (center), which is a long spiraling tube, divided into three compartments. The scala vestibuli (SV) and scala tympani (ST) contain perilymph (138 mM Na+, 6.9 mM K+) and the scala media (SM) contains endolymph (2 mM Na+, 145 mM K+).^[^ ^12^ ^]^ There are three main sites in the cochlea that can cause sensorineural hearing loss when damaged: 1) the stria vascularis (magnified left) which maintains the cochlear electrochemical gradient by recycling potassium ions (K+) and provides a protective blood‐labyrinth barrier, 2) the organ of Corti (magnified upper right), which contains the hair cells that convert sound into an electrochemical signal, and 3) the spiral ganglion (SG) neurons (magnified lower right), which convey auditory signals via the cochlear nerve to the brainstem. Type I spiral ganglion neurons receive auditory signals from the inner hair cells and convey these signals via the cochlear nerve to the brainstem, and type II neurons provide efferent signals from the brain. Type I neurons primarily innervate inner hair cells (IHCs) and type II neurons innervate outer hair cells (OHCs) (reviewed in ref. [13]).

Dysfunction at any point in the auditory pathway can cause hearing loss. However, the preponderant cause of hearing loss is damage within the inner ear, to the sensory hair cells, auditory neurons, the synapses between the sensory cells and auditory neurons, or the stria vascularis (**Figure** **1**) (reviewed in ref. [6]). Audiometric threshold measurements—the gold standard for hearing evaluation—cannot pinpoint which aspect of the inner ear has been damaged.^[^ ^7^ ^]^ Moreover, the inner ear cannot be readily accessed for clinical investigation because the ear is enclosed in the temporal bone. As a result, the exact cause of an individual's hearing loss remains elusive, which is a significant roadblock for treatment.^[^ ^8^ ^]^ On the other hand, blood‐based analyses for alternate conditions have facilitated rapid and early diagnoses by detecting disease‐specific biomarkers. For example, in the case of diabetes, blood glucose is monitored using a glucometer.^[^ ^9^ ^]^ This diagnostic approach presents a functional technology (i.e., a biosensor) already capable of rapid detection for inner ear biomarkers, once those markers are described.^[^ ^10^ ^]^ Biosensor technology combines a molecular capturing mechanism based on antibody, enzyme, or synthetic DNA, with an optical, mass, or electrochemical readout strategy to report the level of a specific biomarker as a pathological indicator of disease. Engineering advancements in biosensing technologies have enabled “rapid point‐of‐care (POC) diagnostics,” that provide sensitive detection and quantification of biomarkers in a timely manner at the point of need.^[^ ^11^ ^]^ Thus, efforts are required to identify inner ear biomarkers that specifically indicate physiological changes within the ear, to provide rapid and accurate diagnosis of site‐specific hearing loss.

## 2 DNA and RNA Expression Profiles Associated with Hearing Loss

### 2.1 Protein‐Coding Genes

Various forms of gene and protein analysis, from sanger sequencing, exome sequencing, full genome sequencing, and RNA transcriptional profiling have been used to identify hundreds of genes and mutations associated with hearing loss (https://hereditaryhearingloss.org/). In most cases, these mutations cause congenital deafness, and the individual's diagnostic odyssey begins with multigene panel testing that screens for mutations in common deafness‐associated genes.^[^ ^14^ ^]^ Alternatively, whole‐genome sequencing is becoming more affordable and improving genetic diagnoses for individuals carrying less common or novel hearing loss‐associated mutations.^[^ ^2^ ^]^ However, there are notable instances where a POC test for a hearing loss‐associated mutation could radically change an individuals’ treatment plan. For example, a well‐characterized mutation in the mitochondrial 12S ribosomal RNA gene (m.1555 A to G) renders an individual particularly susceptible to aminoglycoside ototoxicity. The mutation occurs in the general population at a rate of ~0.2%.^[^ ^15^ ^]^ However, it is found in 10–34% of people with aminoglycoside‐induced ototoxicity (reviewed in ref. [16]). Therefore, a rapid test for this mutation would inform clinicians on the increased likelihood of ototoxic outcomes, potentially encouraging another course of treatment or an improved counseling plan for the treated individual regarding ototoxic outcomes.

General mitochondrial activity is also critical for maintaining ear‐health. Mitochondrial DNA (mtDNA) is the circular genome component of intracellular mitochondria. It can be detected extracellularly as cell‐free DNA (cfDNA) which is released during cellular apoptosis or necrosis and then circulates in bodily fluids. It has been well documented that mtDNA mutations can alter mitochondrial function, causing excessive reactive oxygen species production and enhanced apoptotic gene expression.^[^ ^17^ ^]^ Recent studies have identified a number of mtDNA mutations, including the m.1555 A to G variant, that are associated with unique aetiologies, ranging from inherited, or aminoglycoside‐induced hearing loss, to age‐related hearing loss.^[^ ^18^ ^]^ Moreover, Falah et al. observed lower median mtDNA copy number relative to the nuclear genome in blood samples from individuals with symmetric, bilateral, age‐related hearing impairment, when compared to healthy controls (based on audiogram analysis), which was associated with the degree of hearing impairment.^[^ ^19^ ^]^ Thus, it is clear that quantification of mtDNA copy number could serve as a potential cell‐free biomarker for early screening of age‐related hearing loss. But further research is still required to ascertain whether mtDNA quantity correlates with mitochondrial activity in the ear.

Protein coding genes may also be useful biomarkers for monitoring the progression of age‐related hearing loss. For example, a study of 52 individuals with age‐related hearing loss and 29 non‐hearing impaired individuals demonstrated that the expression of both pro‐apoptosis Bcl‐2 antagonist killer (BAK1) and apoptosis regulator Bcl‐2 (BCL2) are upregulated in blood samples taken from individuals with hearing loss.^[^ ^20^ ^]^ Specifically, the BAK1:BCL2 ratio was significantly elevated in the group with age‐related hearing loss in correlation with its degree of severity. Therefore, this ratio may be routinely tested to detect the onset and progression of age‐related hearing loss. However, a larger cohort study is required to clarify the pathogenic range of BAK1:BCL2. Beyond BAK1 and BCL2, it is likely that additional genomic targets will be identified for evaluation as potential circulating biomarkers for hearing loss.

### 2.2 Circulating MicroRNAs

Small single‐stranded non‐coding RNAs of 19–25 nucleotides, known as microRNAs (miRNAs) have now been identified in individuals with hearing loss. These non‐coding RNAs can regulate the expression of protein‐coded genes by controlling or preventing the translation of their target mRNA into a protein (reviewed in ref. [21]). Circulating miRNAs are good candidates for blood‐based POC testing because they are cell‐free and stable in many biological fluids, making them easy to detect and measure.^[^ ^22^ ^]^

One of the first miRNAs to be associated with hearing loss was miRNA‐96, when a mutation in the miRNA‐96 seed region was associated with non‐syndromic‐autosomal dominant hearing loss (reviewed in ref. [23]). Subsequently, Mencia et al. demonstrated that single‐base mutations of the MiR‐96 seed region interfere with the miRNA‐96‐mRNA binding process, causing miRNA‐96 to bind incorrectly with both target and off‐target mRNA. This in turn reduces the silencing of miRNA‐96 target proteins and interferes with the translation of other proteins.^[^ ^24^ ^]^ In particular, miRNA‐96 dysfunction causes downregulation of SLC26a5, a gene responsible for prestin synthesis, which consequently causes a shortening of the outer hair cells and results in their degeneration.^[^ ^25^ ^]^ These observations regarding miRNA‐96, a member of the sensory tissue‐specific miRNA‐183 cluster family encouraged further research regarding miRNAs in the ear.^[^ ^26^ ^]^ Subsequently, the miRNA‐183 family (miRNAs‐183, ‐96, and ‐182) was found to be down‐regulated in the cochlea of mice exposed to noise that induces hearing loss.^[^ ^27^ ^]^ In addition, up‐regulation of circulating miRNA‐16‐5p, miRNA‐24‐3p, miRNA‐185‐5p, and miRNA‐451a has been identified in the whole blood of individuals with noise‐induced hearing loss (reviewed in ref. [28]). Therefore, these miRNAs are potential biomarkers that may be monitored in people who are occupationally exposed to loud noise.

Changed miRNA expression has also been observed for age‐related hearing loss, with miRNA‐34a expression increased in the cochlea, auditory cortex, and plasma of aged mice.^[^ ^29^ ^]^ Subsequent human studies also demonstrated that plasma miRNA‐34a levels were higher in 24 individuals with age‐related hearing loss when compared to 58 normal hearing controls, indicating that plasma miRNA‐34a levels are robustly associated with hearing loss. In addition, miRNA‐29a and miRNA‐124 expression levels have been associated with age‐related hearing loss in mice, but not in humans.^[^ ^29^ ^]^ This finding was supported by a small study of people with mitochondrial disease and sensorineural hearing loss, which recapitulated that miRNA‐34a and miRNA‐29b were consistently upregulated in the plasma of individuals with hearing loss, whereas miRNA‐29a was not.^[^ ^30^ ^]^

Overall, tens of microRNAs have been identified in mouse models and human populations with age‐related, sudden onset, or noise‐induced hearing loss.^[^ ^21^ ^]^ However, further evaluation in large human‐based research studies is required to ascertain which of these miRNAs can be used as robust biomarkers of hearing loss. Notably, miRNAs may be able to provide very specific information regarding damage in the ear because of their cell‐specific expression patterns. Moreover, it is possible that the manipulation of these miRNAs will be utilized to mitigate hearing loss outcomes in the future.

## 3 Proteomics and the Inner Ear

To date, few proteins specific to the ear have been identified. However, some proteins that are highly expressed in the ear have been characterized as having unique roles in the development and maintenance of specific inner ear cell types.^[^ ^31^ ^]^ Notably, few of these proteins have been identified as potential blood‐circulating biomarkers for inner ear disorders (**Table** **1**). This may be due to the blood‐labyrinth barrier preventing proteins from passing from inner ear into the blood stream, or it may simply stem from limited research in the area. Only one protein, prestin, has been tested as a circulating biomarker of hearing loss, associated with the damage of inner ear outer hair cells.^[^ ^32^ ^]^

**Table 1:** Protein biomarkers primarily expressed in the inner ear. Online Mendelian Inheritance in Man (OMIM) reference numbers are given for use in https://www.omim.org/. Protein characteristics are as described in https://www.genecards.org/

Protein	Location	Number of amino acids	Weight [kDa]
Tectorins (alpha: OMIM 602574) (beta: OMIM 602653)	Tectorial membranes	Alpha: 2155 Beta: 329	Alpha: 239.527 Beta: 36.956
Otogelin (OMIM 604487)	Acellular membranes (otoconia and cupula of the vestibular organs)	2925	314.794
Otoancorin (OMIM 607038)	Interface of the sensory epithelia and the acellular gel overhead	1153	128.533
Prestin (OMIM 604943)	Outer Hair Cells	744	81.264
Otoconin‐90/95 (OMIM 601658)	Otoconial membrane	477	51.728
Otolin‐1	Otoconial membrane	477	49.422
Cochlin (OMIM 603196)	Nerve fibers between auditory ganglion and sensory epithelium	550	59.483

### 3.1 Circulating Inner Ear Proteins

Prestin, a membrane transport protein encoded by solute carrier anion transporter family 26 member 5 (SLC26A5), is a relatively small protein (8–12 nm diameter). It is bullet‐shaped, and has the ability to enter the blood by crossing plasma membranes in the ear.^[^ ^33^ ^]^ Prestin is highly expressed in the lateral membrane of cochlear outer hair cells and was therefore predicted by Parham and Dyhrfjeld‐Johnsen to be an indicator of outer hair cell damage when found in the blood. In a proof‐of‐concept experiment, Parham and Dyhrfjeld‐Johnsen demonstrated that prestin could be detected by Enzyme‐linked Immunosorbent Assay (ELISA) in the blood of Wistar rats, and that prestin‐blood levels were 56% lower in rats two weeks after noise exposure.^[^ ^32^ ^]^ Whilst further research is required to evaluate prestin as a potential biomarker of hair cell damage in humans, the Parham & Dyhrfjeld‐Johnsen study demonstrates that it may be possible to use prestin as a biomarker for identifying the earliest stages of outer hair cell damage. Notably, a substantial development regarding the analysis of prestin as a potential biomarker is the recent design of a biosensor capable of detecting this protein.^[^ ^10^ ^]^ The biosensor utilizes a DNA‐based immunoassay immobilized on nanostructured electrodes and is capable of detecting low picomolar concentrations of proteins in whole blood. This biosensor can be tailored to target almost any protein biomarker, and as nanostructure technology continues to improve, so too will the sensitivity of future biosensors.

### 3.2 Inflammation‐Related Proteins

Inflammation has been heavily associated with noise and ototoxic drug‐induced inner ear damage, by the release of pro‐inflammatory cytokines, chemokines, and reactive oxygen species (ROS) (reviewed in [34]). In the murine cochlea, intense noise exposure causes elevated levels of pro‐inflammatory proteins and cytokines, including intracellular adhesion molecule‐1 (ICAM‐1), vascular cell adhesion molecule‐1 (VCAM‐1), inducible nitric oxide synthase (iNOS), interleukin (IL)‐1*β* (IL‐1*β*), tumor necrosis factor‐alpha (TNF‐*α*), IL‐6, chemokines (CCL2), and intracellular adhesion molecules (ICAM‐1).^[^ ^35^ ^]^ Recently, increased levels of the chemokine (C‐X‐C motif) ligand 1 (CXCL1) were identified in the mouse inner ear perilymph within six hours of noise exposure.^[^ ^36^ ^]^ Furthermore, high levels of activated caspase‐1, IL‐1*β*, IL‐18, and nucleotide‐binding domain (NOD)‐like receptor protein 3 (NLRP3) were observed in the inner ear of aged mice. Analysis of these biomarkers was limited to perilymph sampling and they have not been assessed in blood samples.^[^ ^37^ ^]^ However, elevated plasma levels of soluble tumor necrosis factor receptor‐2 (TNFR‐2) have been associated with an increased prevalence of self‐reported hearing loss in women over 60 years old.^[^ ^38^ ^]^ In addition, increased levels of C‐reactive protein (CRP) and IL‐6, as well as higher white blood cells counts (particularly neutrophils) has been observed in blood samples collected from individuals with age‐related hearing loss.^[^ ^39^ ^]^ Taken together, these studies indicate that inflammation is an important and measurable indicator of progressive hearing loss. However, it is important to note that biomarkers of inflammation are not particularly specific to cell types in the ear, or the ear at all. Therefore, further research is needed to delineate how inflammation‐associated proteins in the blood can be used to indicate disease progression in the ear.

## 4 Inner Ear Metabolites

Profiling of the auditory metabolome is an emerging area of research that may provide insight regarding the health of the inner ear. Metabolites are very small molecules (<1.5 kDa) and their levels may reflect the functional status of the inner ear.^[^ ^40^ ^]^ Metabolites are particularly useful as biomarkers because they are stable in media, easy to measure, and present in a variety of bodily fluids, cells, and tissues. However, as hearing disorders are localized to the inner ear, interpreting inner ear metabolomic data may require comparisons of metabolite levels between the inner ear fluids and either whole blood or cerebrospinal (CSF) fluid. This would require knowledge of normal and pathological ranges that are yet to be defined in the ear, and would necessitate invasive fluid collection.^[^ ^41^ ^]^ Nevertheless, notable observations have been made regarding metabolites in the damaged ear.

### 4.1 Rodent Metabolites

In mice, 220 metabolites have been identified using whole mouse inner ear tissues, with 40 metabolites showing a significant change after noise‐induced trauma.^[^ ^42^ ^]^ Metabolite levels changed relative to the level of acoustic trauma (i.e., exposure to louder sounds, or duration of exposure), which indicates that metabolites may be useful for assessing the degree of damage caused by noise exposure. For example, glutamate and aspartate levels were increased in the ears of noise‐exposed mice. Glutamate is the main afferent neurotransmitter in the auditory system, mediating neurotransmission between inner hair cells and afferent spiral ganglion neurons. Whereas aspartate is a principal excitatory transmitter in hair cells, supporting cells, and nerve fibers.^[^ ^42^ ^]^ Likewise, a number of identified metabolites have been associated with specific areas in the ear, which may assist in identifying the specific cause of an individual's hearing loss.

In guinea pigs, 77 metabolites have been compared between the inner ear fluid and plasma of noise‐exposed and control animals.^[^ ^43^ ^]^ Ascorbic acid, fructose, galactosamine, inositol, pyruvate + oxaloacetic acid, and meso‐erythritol levels were significantly higher in the inner ear fluid than in plasma, while phosphate, valine, glycine, glycerol, ornithine, glucose, citric acid + isocitric acid, mannose, and trans‐4‐hydroxy‐l‐proline were lower. Significant changes were observed in ten of the inner ear metabolites subsequent to noise exposure, (3‐hydroxy‐butyrate, glycerol, fumaric acid, galactosamine, pyruvate+ oxaloacetic acid, phosphate, meso‐erythritol, citric acid+ isocitric acid, mannose, and inositol).^[^ ^43^ ^]^ However, of these ten metabolites, citric acid + isocitric acid was the only one to show a significant change in plasma.^[^ ^43^ ^]^ This finding may highlight the effect of the inner ear blood barrier, and the challenges of identifying biomarkers capable of crossing from the inner ear into the blood.

### 4.2 Human Metabolites

Metabolite investigations have now progressed from animal models to humans, with Mavel et al. identifying 98 metabolites in 23 perilymph and cerebrospinal fluid samples taken from humans with bilateral sensorineural hearing loss during cochlear implantation.^[^ ^44^ ^]^ The identified metabolites in this study included amino acids, carboxylic acids, and derivatives such as lactate, carnitine, trigonelline, and creatinine. Interestingly, the overall perilymph metabolic signatures correlated with the duration of each individuals hearing loss.^[^ ^44^ ^]^ Of the 98 metabolites identified, 15 were common between humans and guinea pigs. However, the intensity of mass spectrometry signals for creatinine was very high in human perilymph, whereas lactate had the greatest intensity in both humans and guinea pigs.^[^ ^44^ ^]^ Further human cohort studies are now required to elucidate which metabolite biomarkers are useful indicators of human inner ear health and at which age they may be most useful (as the perilymph metabolomic profile is different between children ≤12 years old and those >12 years of age^[^ ^45^ ^]^).

## 5 POC Detection of Biomarkers

**Figure 2:**
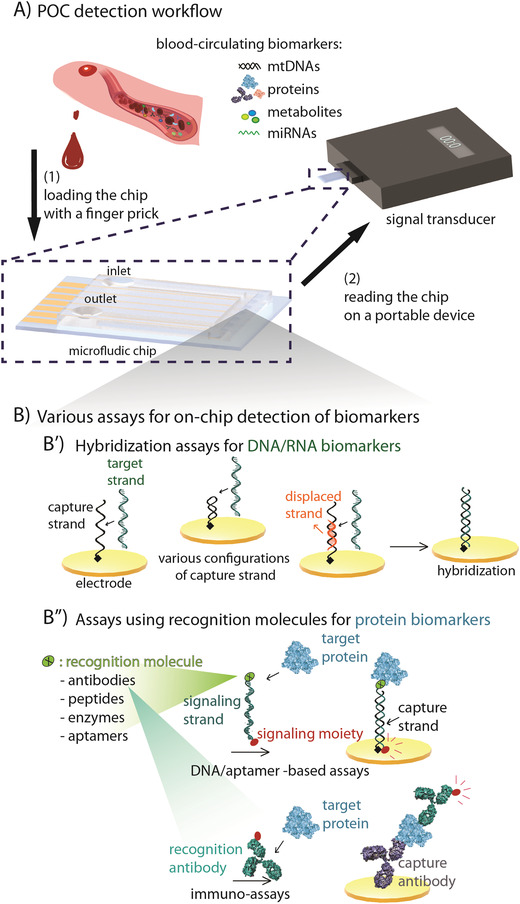
Biosensor detection at the POC. A) Detection workflow: (1) Blood from a finger prick is loaded onto a microelectronic chip (magnified left) that detects and reports biomarker levels to (2) a portable device. B) Assays can use recognition strategies such as synthetic oligonucleotide sequences, antibodies, peptides, or aptamers. B′) Assays for detecting genomic targets often use capture DNA strands (black) that are chemically immobilized on the surface of the electrode. Capturing the genomic target will result in conformational or configurational changes. The displaced strand (orange) is a short strand being displaced by a target strand which is longer, due to the higher affinity of the target strand for hybridization. B″) Assays for the detection of non‐genomic targets via (B″, top) synthetic oligonucleotides, or (B″, bottom) chemically immobilized antibodies that are conjugated to a target recognition molecule and carry the redox‐active moiety (red dot; generating electron) for signal readout upon hybridization or binding.

POC diagnostic strategies facilitate earlier diagnoses and therapeutic interventions, which can limit disease progression and improve patient outcomes.^[^ ^46^ ^]^ Conversely, conventional molecular detection methods, such as polymerase chain reaction (PCR) or ELISA are still based on multiple‐step, reagent‐intensive, and time‐consuming processes that require expensive infrastructure. POC technologies are capable of rapid biomarker detection in complex media (e.g., whole blood) at remote locations, mostly by combining biomarker recognition strategies (e.g., through antibody‐antigen or aptamer‐protein interactions) with on‐chip biosensing innovations to produce a specific signal readout^[^ ^47^ ^]^ (reviewed in ref. [48]). Notably, electrochemical biosensors become more attractive as they combine the biorecognition interactions with an electrochemical signal readout to provide process simplicity, signal specificity, and rapid turnaround time, in a portable multiplexing (multiple simultaneous analyses in a sample) fashion for POC detection (**Figure** **2**) (reviewed in ref. [49]). In this section, we discuss important design aspects of electrochemical biosensors for detecting potential biomarkers of hearing loss.

### 5.1 Detection of Genomic Biomarkers

Genomic biomarkers constitute oligonucleotide sequences that are conventionally recognized via sequencing techniques. For genetic sequencing, tissue samples must be collected and transported to an accredited lab where DNA or RNA is extracted and prepared for analysis. This process can take hours to weeks depending on the specific analysis requested and the availability of trained personnel, equipment, and reagents. Next generation sequencing also requires bioinformatic data analysis, due to the complexity of data provided (reviewed in ref. [50]). Furthermore, once variants of interest are identified, the gold standard validation method includes polymerase chain reaction (PCR) and sanger sequencing. This is a reagent‐intensive laboratory‐based process, in which the extracted nucleic acids are amplified and fluorescently tagged to facilitate highly accurate sequencing^[^ ^51^ ^]^

Alternatively, specific genetic mutations or circulating miRNA's can be detected using a biosensor (**Table** **2**). Oligonucleotide sequences are usually detected using synthetic DNAs (the complementary sequence) immobilized on the biosensor surface. Once the target is bound to the sensor surface, hybridization induces conformational or configurational changes that result in the transfer of a biomolecule to the conductive surface, resulting in activation of redox elements that produce electrons, generating a detectable signal (Figure 2B).^[^ ^52^ ^]^ Sensor sensitivity relies on the affinity of target hybridization occurring in the vicinity of the electrode surface. There are still limitations regarding the electrochemical biosensing platforms for DNA and RNA POC‐detection, including ultra‐low sample concentrations (particularly in a finger‐prick volume), biomarker/biosensor degradation during the detection time‐frame, and the low specificity of oligonucleotide sequences for accurately identifying single point mutations (reviewed in ref. [53]).

**Table 2:** Examples of electrochemical biosensors that detect miRs in blood

Detection platform	Detection target	Specificity and mismatches	Detection time	Detection range/ Detection limit	Detection media	Publication year
Electrode: flat gold E/chitosan/origami‐ssDNA	miR‐21: 5′‐UAG CUU AUC AGA CUG AUG UUG A‐3′	vs. (1)^δ^: 5′‐UAG CUU AUC AGA CUG AUG UUG **C**‐3′ (2)^δ^: 5′‐UAG CUU AUC **G** GA CUG AUG UUG A‐3′ (3) miR‐192: 5′‐CUG ACC UAU GAA UUG ACA GCC‐3′	N/A	0.1 pM to 10.0 nM/79.8 fM	spiked into 1% human serum	Shuo Han, 2019^[^ ^65^ ^]^
Electrode: DNA–Au@MNPs Collector: flat gold E using a magnet	miR‐21: 5′‐UAG CUU AUC AGA CUG AUG UUG A‐3′	vs. (1)^δ^: 5′‐UAG CUU AUC A **A** A CUG AUG UUG A‐3′	30 min	10 aM to 1 nM	(1) spiked unpurified serum (2) spiked 50% blood	R. Tavallaie, et al., 2018^[^ ^66^ ^]^
Label: Fe3O4/CeO2 @Au MNPs Electrode: GCE electrodeposited AuNPs	miR‐21: 5′‐UAG CUU AUC AGA CUG AUG UUG A‐3′	vs. (1) miR‐141: 5′‐CAU CUU CCA GUA CAG UGU UGG A‐3′, (2) miR‐155: 5′‐UUA AUG CUA AUC GUG AUA GGG GUU‐3′ (3) miR‐199: 5′‐CCC AGU GUU CAG ACU ACC UGU UC‐3′	100 min	1 fM to 1 nM/0.33 fM	Spiked into 10‐fold‐diluted human serum	Sihan Liu, et al., 2018^[^ ^67^ ^]^
Labels: 1) Au/TMC/Fe3O4, 2) CdSe@CdS/TMC/Fe3O4 Electrode: PTh/rGO/SPCE	(1) miR‐106a: 5″‐AAA AGU GCU UAC AGU GCA GGU AG‐3″ (2) miR let‐7a: 5″‐UGA GGU AGU AGG UUG UAU AGU U‐3″	vs. (1) miR‐15a: 5″‐UAG CAG CAC AUA AUG GUU UGU G‐3″ (2) miR‐21: 5″‐UAG CUU AUC AGA CUG AUG UUG A‐3″ (3) miR‐200c: 5′‐CGU CUU ACC CAG CAG UGU UUG G‐3	N/A	Serial dilutions of targets: 0.1 fM‐5 pM/ miRNA‐106a: 0.06 fM miRNA let‐7a: 0.02 fM	Spiked human blood plasma	Maryam Daneshpour, et al., 2018^[^ ^68^ ^]^
Labels: 1) biotin‐MB1‐AuNPs, 2) biotin‐MB2‐AgNPs Electrode: Neutravidin/GCE	(1) miR‐21: 5′‐UAG CUU AUC AGA CUG AUG UUG A‐3′ (2) miR‐141: 5′‐UAA CAC UGU CUG GUA AAG AUG G‐3′	vs. (1) simultaneous detection of miR‐21 and miR‐141 (2) miR‐205: 5′‐UCCUUCAUUCCACCGGAGUCUGU‐3′ (3) miR‐221: 5′‐AGC UAC AUU GUC UGC UGG GUU UC‐3′	N/A	miRNA‐21: 0.5–1000 pM/0.3 pM miRNA‐141: 50−1000 pM / 10 pM	spiked serum sample	Sawsen Azzouzi, et al. 2019^[^ ^69^ ^]^
Amplification: combined CESA with template‐free DNA extension reaction Electrode: flat gold E	miR‐196a: 5′‐UAG GUA GUU UCA UGU UGU UGG G‐3′	vs. (1)^δ^: 5′‐UAG GUA GUU ACA UGU UGU UGG G‐3′ (2) miR‐196b:5′‐UAG GUA GUU UCC UGU UGU UGG G‐3′ (3): 5′‐UAG CUU AUC AGA CUG AUG UUG A‐3′	60 min	0.05 fM to 50 pM/15 aM	Spiked plasma	Jing Guo, et al., 2018^[^ ^70^ ^]^

E: electrode; GCE: glassy carbon electrode; SPCE: screen printed carbon electrode. Au@MNPs: gold‐coated magnetic nanoparticles; Au MNPs: gold magnetite nanoparticles; AuNPs: gold nanoparticles; AgNPs: silver nanoparticles; TMC: N‐trimethylchitosan (TMC) polymer; PTh: Polythiophene; rGO: reduced graphene oxide; CESA: cyclic enzymatic signal amplification; biotin‐MB: biotinylated molecular beacon; ssDNA: single‐stranded DNA; ^δ^single‐mismatch base‐pair: bold‐underlined letter.

However, advanced nanostructured electrodes are improving biosensor sensitivity and specificity.^[^ ^54^ ^]^ In particular, target recognition and binding efficiency have been improved by nanostructuring, which increases the probe‐target attachment area, accelerating electron transfer, and controlling the kinetics of redox reactions. The use of nanostructured electrodes may facilitate target detection in smaller sample volumes. However, this also requires that efficient electron transfer is maintained despite the significantly reduced size of electrode structures. Examples where nanostructures are currently used, include gold nanostructured microelectrodes (Au‐NMEs),^[^ ^55^ ^]^ and ultra‐conductive carbon‐based materials, such as carbon nanotubes and graphene nanosheets (reviewed in ref. [56]).

### 5.2 Detection of Protein Biomarkers

The detection and quantification of protein biomarkers can be difficult, particularly in complex media. Conventionally, protein quantification requires multistep and reagent‐intensive methods such as ELISAs^[^ ^57^ ^]^ and Western blots.^[^ ^58^ ^]^ Notably, western blots are labor intensive and not particularly sensitive for quantifying protein levels. Whereas ELISAs have adequate sensitivity, but require greater sample preparation, including accurate serial dilution and washing steps, multiple reagent incubations, and antibody detection. Conversely, several electrochemical biosensors have been developed that are capable of accurately detecting and quantifying protein levels in a one‐step or a one‐pot process (**Table** **3**). For these biosensors, elements of protein biorecognition include recombinant antibodies, synthetic peptides, enzymes, and aptamers that are highly specific to the protein of interest (Figure 2B).^[^ ^59^ ^]^ These biorecognition elements are typically used within a surface‐immobilized assay, such as a DNA‐based assay with DNA‐DNA hybridization that transfers a conjugated protein to the electrode surface (Figure 2B′), an aptamer‐based assay with the aptamer‐binding protein driving the target to the electrode surface, or an immunoassay with surface antibody‐protein interactions on the electrode (Figure 2B″). However, all of these platforms are limited by a number of factors in their ability to accurately detect biomarkers, such as the binding affinity of the recognition molecule, signal instability in whole blood‐ particularly when a redox reporter is initially placed on the electrode surface;^[^ ^60^ ^]^ and the low concentration of biomarkers in a small sample volume, which decreases the overall sensitivity of the assay.^[^ ^53a^ ^]^ Therefore, the use of nanostructured surfaces will likely be needed to design biosensors that are sensitive enough to detect inner ear‐specific proteins in circulating blood.

**Table 3:** Examples of electrochemical biosensors that detect proteins in blood

Detection platform	Detection target	Recognition element	Detection time	Detection range/Detection limit	Detection media	Publication year
Flat gold E DNA‐based	Anti‐HIV antibodies	24 kDa HIV p24 antigen	N/A	Few nanomolar	Minimally diluted blood serum	Di Kang, et al., 2018^[^ ^71^ ^]^
Ag SPE DNA‐based sensor	(1) Cetuximab (2) Anti‐HIV (3) Anti‐Dig (3) Anti‐DNP (4) Anti‐HP	Antigen‐conjugated single‐stranded DNAs	30 min incubation	Low nanomolar detection limit	90% bovine blood serum	Sara Bracaglia, et al., 2021^[^ ^72^ ^]^
Flat gold E DNA‐/aptamer‐based	Thrombin	Thrombin aptamer	80 min	2 pM – 20 nM / 0.76 pM	Diluted human serum	J Zhu, et al., 2018^[^ ^73^ ^]^
Flat gold E: Au‐GO/SA‐aptamer–AQ/MB/Fc Aptamer‐based sensor	(1) VEGF (2) IFN‐*γ* (3) TNF‐*α*	Biotinylated (1) VEGF aptamer (2) IFN‐*γ* aptamer (3) TNF‐*α* aptamer	30 min aptamer‐reaction time	(1) VEGF: 5–300 pg mL^−1^ (2) IFN‐*γ*: 5–300 pg mL^−1^ (3) TNF‐*α*: 5–200 pg mL^−1^/5 pg mL^−1^	Spiked human serum and artificial sweat	Zhuping Shen, et al., 2021^[^ ^74^ ^]^
Poly(o‐phenylenediamine) nanosphere‐GCE Immunosensor	CEA	horseradish peroxidase‐labeled polyclonal rabbit anti‐human CEA antibody	20 min	0.01 to 60 ng mL^−1^/3.2 pg mL^−1^	human serum	Ti‐Sen Xu, et al., 2015^[^ ^75^ ^]^
AuNPs‐modified GCE Immunoassay	Prion protein	Antibody‐antigen	Over 90 min	0.5 pg mL^−1^ ‐ 100 ng mL^−1^/0.38 pg mL^−1^	human serum	Xiaoyu Li, et al., 2018^[^ ^76^ ^]^
Flat gold E Protein receptor‐based	Synthetic triacylated lipopeptide Pam3CSK4	Two combined toll‐like receptors	N/A	5 µM (7.5 µg mL^−1^)	Spiked HEPES buffer	Zhe She, et al., 2018^[^ ^77^ ^]^
Antibody/d‐BSA/rGO thin film E Immunosensor	Cortisol	Anti‐cortisol antibody probes	N/A	10 pM–100 nM/10 pM	human saliva	Kwang Su Kim, et al., 2016^[^ ^78^ ^]^
g‐C_3_N_4_@Au@Fc‐NH_2_ DNA‐based	PDGF	DNA hydrogel blocker formed by target‐induced homogeneous entropy catalytic amplification	100 min	0.01 pM–10 nM/3.5 fM	human blood serum	Yuanyuan Chang, et al., 2018^[^ ^79^ ^]^
Flat gold E DNA‐based	Anti‐digoxigenin, streptavidin	digoxigenin, biotin	10 min	1 nM–100 nM/10 nM	Spiked whole blood	Sahar S Mahshid, et al., 2015^[^ ^80^ ^]^
Au NMEs DNA‐based immunoassay	(1) RANTES: (2) MDC (3) LAP	(1) anti‐RANTES (2) anti‐MDC (3) anti‐LAP	40 min	10 pg mL^−1^ – 10 ng mL^−1^/10 pg mL^−1^	HSC fed‐batch bioreactor culture	W. Zhou, et al., 2017^[^ ^81^ ^]^

E: electrode; SPE: screen printed electrode; GO: graphene oxide; rGO: reduced graphene oxide; GCE: glassy carbon electrode; NME: nanostructured microelectrodes; AuNPs: gold nanoparticles; AQ: anthraquinone (redox moiety); MB: methyl blue (redox moiety); Fc: ferrocene (redox moiety); RANTES: regulated on activation, normal T cell expressed, and secreted; MDC: macrophage‐derived chemokine; LAP: latency‐associated peptide; HSC: hematopoietic stem cells; PDGF: platelet‐derived growth factor; CEA: carcinoembryonic antigen; VEGF: Vascular endothelial growth factor; IFN‐*γ*: interferon‐*γ*; TNF‐*α*: Tumor necrosis factor‐*α*.

### 5.3 Detection of Metabolite Biomarkers

Currently, the detection of metabolites requires multiple steps, including metabolite separation (chromatography), identification, and quantification (often performed by mass spectrometry analysis, with read‐out interpretation using specialized software).^[^ ^61^ ^]^ Mass spectrometry is a labor intensive procedure requiring ionization, separation based on mass per charge, and detection of the current of the separated ions.^[^ ^62^ ^]^ Replicating these steps in a biosensor platform is difficult. However, a number of mostly enzymatic‐based platforms have been developed that can accurately detect specific metabolites (**Table** **4**). An important limitation for detecting metabolites of localized disease (such as hearing loss) is that the sample used for analysis must be collected from fluid in direct contact with the affected organ. This is because metabolites in the blood or CSF can derive from any perfused tissue in the body.^[^ ^11^, ^63^ ^]^ Therefore, the metabolomic profile of inner ear fluid may be more useful for analysis than whole blood samples for detecting inner ear disorders. However, the inner ear fluid cannot be accessed non‐invasively. In this respect, the discovery of metabolites that are specifically associated with ear health and found in the blood would be particularly useful. Thus, further research is required to establish whether inner ear‐specific metabolites can be detected in the blood and to define normal and pathological levels.^[^ ^43^ ^]^

**Table 4:** Examples of electrochemical biosensors for detection of metabolites in biological fluids

Detection platform	Detection target	Specificity and recognition element	Detection time	Detection range/Detection limit	Detection media	Publication year
PB‐PPD‐LOx E Enzymatic platform	Salivary lactate concentrations	LOx enzyme	10 min	0.1–0.5 mM / 0.202 µA mM^−1^	Human saliva samples	Jayoung Kim, et al., 2014^[^ ^82^ ^]^
PtNPs‐modified CPH GCE Enzymatic platform	(1) uric acid (2) cholesterol (3) triglycerides	PANI/PtNPs/enzyme hybrid film (1) UOx (2) ChEt/ChOx (3) LIP/GK/GPO	≈3 s	(1) uric acid: 0.07–1 mM (2) cholesterol: 0.3–9 mM (3) triglycerides: 0.2–5 mM	PBS	Lanlan Li, et al., 2015^[^ ^83^ ^]^
AuNPs/PB flat gold E Enzymatic platform	(1) glucose (2) lactate	(1) GOx enzyme (2) LOx enzyme	N/A	(1) glucose: 26.31 µA/mM⋅cm (2) lactate: 1.49 µA/mM⋅cm	(1) Artificial sweat (2) Human sweat (collected after a period of constant workload)	Murat A. Yokus, et al., 2020^[^ ^84^ ^]^
Thin‐film gold E *Matrix*: collagen I hydrogel interface aptamer‐based sensor	ATP	ATP Aptamer	Real‐time	1–10 µM/2.5 µM	Cell culture media	Mirelis Santos‐Cancel, et al., 2019^[^ ^85^ ^]^

E: electrode; GCE: glassy carbon electrode; CPH: conducting polymer hydrogels; AuNPs: gold nanoparticles; PtNPs: platinum nanoparticles; PB: Prussian blue; ATP: adenosine triphosphate; PANI: polyaniline; PBS: phosphate buffer solution; UOx: uricase; ChEt/ChOx: cholesterol esterase/cholesterol oxidase; LIP/GK/GPO: lipase/glycerol kinase/glycerol‐3‐phosphate oxidase; PPD: poly‐orthophenylenediamine; LOx: lactate‐oxidase.

## 6 Concluding Remarks

Conventional methods of hearing assessment rely on audiometric measurements that cannot precisely indicate the sites of cellular damage in the inner ear. Whereas biosensor approaches are capable of detecting biomarkers that if specifically identified, could potentially indicate the site of damage, facilitating precision therapy when available and providing a method to monitor the efficacy of treatment.

A number of biomarkers for hearing loss have been identified in blood, including cell‐free DNAs, miRNAs, proteins, and metabolites. However, knowledge of these biomarkers and their usefulness in the clinical setting varies. For example, miRNAs represent the most studied aspect of inner ear transcriptomics, having been associated with noise‐induced and age‐related hearing loss. Alternatively, circulating cell‐free DNA is one of the most readily available biomarkers for rapid analysis and may be very useful for the identification of well‐characterized mutations that impact clinical care. Conversely, there are very few proteomic biomarkers for inner ear disorders. Currently, prestin is the main contender as a biomarker for hearing loss, which is an indicator of outer hair cell damage. Likewise, metabolomics of the inner ear may provide a unique opportunity for understanding the progression of inner ear damage. However, the need for defined “normal” and “pathological” ranges currently limits metabolomic utility. Nevertheless, significant progress has been made toward identifying and understanding what biomarkers are available for evaluating inner ear damage.

Another notable challenge for biosensor platform design, is achieving adequate sensitivity and specificity. The platform must include the most suitable biorecognition element for the target biomarker, and these elements must be able to detect and quantify the biomarker consistently and accurately, within its biological range. Overall, the biosensor must successfully combine surface immobilization, hybridization, bioconjugation, and unique (bio)chemical effects such as steric hindrance to achieve a flawless electrochemical readout. There are also a number of challenges regarding the detection of inner ear‐specific biomarkers that must be considered when evaluating biomarkers for clinical applications, including which biological fluid is most useful for analysis, how much is required, and what is easily accessible (**Table** **5**). However, biosensor design is rapidly evolving to include nanostructure technologies that will improve sensitivity and reduce the required sample volume.

**Table 5:** Challenges for electrochemical biosensor design and detection of inner ear specific biomarkers

	Challenge	Potential solutions
Sample	Blood: Complex mix of proteins from many organs and tissues, not specific to inner ear	Identify biomarkers in blood that are inner ear specific
	CSF: Highly invasive to collect	Utilize blood biomarkers
	Inner Ear Fluid: Highly invasive to collect, likely to cause damage during collection	Utilize blood biomarkers
Target Capture	Low sample quantity or stability	– Design biosensors capable of rapid sample assessment^[^ ^53a^ ^]^ – Utilize microfluidic devices in biosensor design to improve sample‐accessibility^[^ ^53b^ ^]^ – Utilize nanostructure technologies to increase target recognition and binding^[^ ^86^ ^]^
	Identifying a specific protein in a complex sample.	Design recognition elements with high specificity target detection^[^ ^49a^ ^]^
	Identifying point mutations	Detection based on charge transport through DNA recognition molecule^[^ ^87^ ^]^
Electrode Design	Nanostructuring can impact sensitivity	Design high‐curvature nanostructures^[^ ^88^ ^]^
	Limited immobilization efficacy of the capture element	Immobilize biomolecules on surfaces using ligands such as thiol^[^ ^89^ ^]^
	Poor sensitivity of the readout strategy when using whole blood	Utilize redox active molecules that are stable in blood

Notably, optical‐based diagnostics are also improving, which may provide opportunities for completely non‐invasive biomarker detection (reviewed in ref. [64]). However, optical‐based diagnostics have not yet progressed to the same sensitivity as fluid‐based biosensors and none have been tested that target inner ear biomarkers. Furthermore, fluid‐based biosensors will likely be required to adequately characterize biomarkers of inner ear disease before optical detection of inner ear‐specific biomarkers can be investigated.

To date, only one report describes the use of an advanced biosensor approach for detecting inner ear biomarkers in whole blood.^[^ ^10^ ^]^ However, this platform can be advanced for more accurate detection within biological ranges and be adapted to identify most blood‐circulating proteins. Therefore, biosensors may become more useful as additional novel inner ear biomarkers are identified in blood, or other bodily fluids.

As precision medicine is rapidly evolving, it is likely that important discoveries in the field of otology will be achieved. This will advance therapeutic approaches for inner ear disorders, which will require novel biomarkers for rapid and accurate diagnosis. Encouragingly, biosensor platform design is now at the forefront of bio‐engineering research, leading to enhanced biosensor sensitivity and specificity for genomic, transcriptomic, proteomic, and metabolomic biomarkers. Ultimately, the identification of robust biomarkers for hearing loss, combined with advancing biosensor design will facilitate rapid diagnostics that can identify the loci of hearing damage. Consequently, clinicians will be able to identify and monitor the progression of an individual's hearing loss and provide a tailored treatment plan.

## Conflict of Interest

The authors declare no conflict of interest.
